# Elderly-Onset Sarcoidosis and the Influence of Age on Clinical Phenotypes: A Multicentre Analysis from the SARCOGEAS Registry

**DOI:** 10.3390/jcm15218355

**Published:** 2026-10-27

**Authors:** María García-González, Carlos Feijoo-Massó, Alejandra Flores-Chávez, Guillem Policarpo-Torres, Ricardo Gómez de la Torre, Begoña de Escalante Yangüela, Jesús Miguel López-Dupla, Cristina Soler-I-Ferrer, Eva Fonseca-Aizpuru, Andrés González-García, Rosana Rouco, Salvador García Morillo, Ana Alguacil, Ana Noblejas-Mozo, Mariona Bonet, María Barrientos, Antonio-J. Chamorro, Borja De Miguel-Campo, Maria Gracia Cruz Caparrós, Miriam Akasbi Montalvo, Alex Mayer-Fuentes, Manuel Ramos-Casals, Pilar Brito-Zerón

**Affiliations:** 1 Department of Internal Medicine, Parc Taulí Hospital Universitari, Institut d’Investigació i Innovació Parc Taulí (I3PT-CERCA), Universitat Autònoma de Barcelona, 08208 Sabadell, Spain; mgarciago01@tauli.cat (M.G.-G.); amayer@tauli.cat (A.M.-F.); 2 Department of Autoimmune Diseases, ICMiD, Hospital Clínic, 08036 Barcelona, Spain; ale.floreschavez@hotmail.com (A.F.-C.); mramos@clinic.cat (M.R.-C.); 3 Department of Internal Medicine, Hospital Universitari Doctor Josep Trueta, IDIBGI, 17007 Girona, Spain; gpolicarpo.girona.ics@gencat.cat; 4 Department of Internal Medicine, Hospital Universitario Central de Asturias (HUCA), 33011 Oviedo, Spain; ricardoagdt@gmail.com; 5 Department of Internal Medicine, Hospital Clínico, 50009 Zaragoza, Spain; bescalantester@gmail.com; 6 Department of Internal Medicine, Hospital Joan XXIII, 43005 Tarragona, Spain; jmlopezdupla.hj23.ics@gencat.cat; 7 Department of Internal Medicine, Hospital Santa Caterina, 17190 Salt, Spain; csoler@comg.cat; 8 Department of Internal Medicine, Hospital de Cabueñes, 33394 Gijón, Spain; evamfonseca@yahoo.es; 9 Department of Internal Medicine, Hospital Ramón y Cajal, 28034 Madrid, Spain; andres_gonzalez_garcia@hotmail.com; 10 Department of Internal Medicine, Hospital CIMA Sanitas, 08034 Barcelona, Spain; rrouco@sanitas.es; 11 Department of Internal Medicine, Hospital Virgen del Rocío, 41013 Sevilla, Spain; salvaymar@gmail.com; 12 Department of Internal Medicine, Hospital Virgen de la Salud, 45004 Toledo, Spain; almoan11@gmail.com; 13 Department of Internal Medicine, Hospital Universitario La Paz, 28046 Madrid, Spain; ananoblejas@hotmail.com; 14 Department of Internal Medicine, Althaia, Xarxa Assistencial de Manresa, 08243 Manresa, Spain; mariona.bonet2@gmail.com; 15 Department of Internal Medicine, Hospital Gregorio Marañón, 28007 Madrid, Spain; maria.barrientos@salud.madrid.org; 16 Department of Internal Medicine, Hospital General Universitario de Salamanca-IBSAL, Universidad de Salamanca, 37007 Salamanca, Spain; ajchamorro@usal.es; 17 Department of Internal Medicine, Hospital 12 de Octubre, 28041 Madrid, Spain; borja.demiguel@gmail.com; 18 Department of Internal Medicine, Hospital General Universitario de Alicante, 03010 Alicante, Spain; grcrca@hotmail.com; 19 Department of Internal Medicine, Hospital Infanta Leonor, 28031 Madrid, Spain; makasbi@hotmail.com; 20 Department of Medicine, Faculty of Medicine and Health Sciences, Universitat de Barcelona, 08036 Barcelona, Spain

**Keywords:** sarcoidosis, elderly-onset sarcoidosis, age at diagnosis, extrapulmonary involvement, multicentre study, sex differences

## Abstract

**Background/Objectives:** To determine the proportion of elderly-onset sarcoidosis (EOS) and to assess the influence of age at diagnosis on pulmonary and extrapulmonary manifestations. **Methods:** Retrospective multicentre study using the Spanish SARCOGEAS registry, including adults with sarcoidosis diagnosed across 36 hospitals. EOS was defined as diagnosis at ≥65 years. Associations between clinical features and age were assessed using multivariable logistic and linear regression models, including sex-stratified analyses. **Results:** Among 2187 patients (mean age 48.6 ± 15.2 years, 61.36% women), 358 (16.37%) had EOS. In multivariable analyses, female sex (OR 1.86; β +5.02 years), White ethnicity (OR 5.43; β +4.85 years), and renal involvement (OR 2.29; β +6.86 years) were independently associated with EOS and older age at diagnosis, whereas cutaneous (OR 0.49; β −3.50 years), articular or bone (OR 0.37; β −3.10 years), and neurological involvement (OR 0.49; β −2.47 years) were associated with non-EOS and younger age at diagnosis. Exploratory sex-stratified analyses suggested additional patterns: in women, calcium metabolism abnormalities were associated with EOS (OR 1.74) and older age at diagnosis (β +4.91 years), and ear–nose–throat involvement was associated with older age at diagnosis (β +5.74 years). Regarding pulmonary involvement, CT-based Scadding stage II was associated with younger age at diagnosis (β −3.75 years) in women and CT-based Scadding stage IV with older age at diagnosis (β +6.57 years) in men. **Conclusions:** EOS accounted for 16.37% of sarcoidosis cases. Older age at diagnosis was associated with specific patterns of organ involvement, which changed gradually across the age spectrum, some of them differing by sex in exploratory analyses. Further studies are needed to confirm these findings.

## 1. Introduction

Sarcoidosis is a heterogeneous multisystem granulomatous disease of unknown cause characterised by the formation of non-caseating granulomas in affected tissues and a highly variable clinical course. Although pulmonary involvement is present in 90% of patients, any organ system may be affected, including the skin, eyes, heart, nervous system, kidneys, liver, and spleen, often in a multiorgan pattern [1,2,3]. Diagnosis is challenging and relies on a compatible clinical and radiological presentation, histological evidence of non-necrotizing granulomas when available, and careful exclusion of alternative granulomatous disorders [4].

Its epidemiology shows striking geographic and ethnic variability. Incidence and prevalence differ widely across regions, with higher incidence rates reported in Northern Europe and among individuals of African ancestry and lower rates in Asian populations [5,6]. Beyond genetic susceptibility, evidence supports environmental and occupational exposures playing an important role in the disease, as well as region of residence and seasonality, in shaping both disease risk and clinical expression [7,8]. 

According to age at diagnosis, sarcoidosis has traditionally been considered a disease of young and middle-aged adults, with peak incidence between the third and fifth decades [9]. However, population-based studies show a bimodal age distribution, with a second peak at older ages, particularly in women [10,11]. More recent registry studies also suggest that the mean age at diagnosis has increased over time [12,13]. Notably, a substantial proportion of cases occur at ≥65 years, supporting specific attention to patients with elderly-onset sarcoidosis (EOS). Although no universally accepted age threshold exists for defining EOS, the cut-off of ≥65 years has been most consistently adopted across the available literature [14,15,16,17,18]. Sex is also an important modifier of the disease, with consistent differences in incidence, clinical presentation, and organ involvement [19]. 

Despite this evolving epidemiology, EOS remains poorly characterised, with most evidence derived from small retrospective or single-centre studies [14,15,16,18,19,20,21,22]. Available data suggest potential differences, with older patients being less likely to exhibit acute inflammatory features, instead presenting with a more insidious and nonspecific onset, a higher burden of comorbidities, and a female predominance. Moreover, organ-specific patterns of involvement have been insufficiently studied, and findings across the few available studies remain inconsistent and heterogeneous [14,15,16,20,21,22].

Nevertheless, several important uncertainties remain. The proportion of EOS in large, unselected populations has yet to be determined. Furthermore, how pulmonary and extrapulmonary manifestations vary between patients with and without EOS and across the age spectrum remains poorly characterised. These gaps are partly attributable to the small sample sizes of available studies, the absence of a universally accepted age threshold for defining EOS, and the inconsistent adoption of validated organ-level assessment instruments such as those proposed by the World Association of Sarcoidosis and Other Granulomatous Disorders (WASOG) organ assessment tool [23], all of which hinder efforts to delineate a distinct EOS phenotype from age-related differences in disease presentation.

In this context, we conducted a nationwide multicentre study using the Spanish SARCOGEAS registry to determine the proportion of EOS (defined as ≥65 years) and to assess the influence of age at diagnosis on the clinical phenotype of sarcoidosis. We analysed EOS and modelled age at diagnosis as a continuous variable to evaluate age-related patterns of organ involvement and to determine whether these differences represent a distinct phenotype or a continuum of disease expression.

## 2. Materials and Methods

### 2.1. Study Design

We conducted a multicentre, observational, retrospective, non-interventional analysis of the SARCOGEAS registry, an ongoing nationwide ambispective registry coordinated by the Autoimmune and Systemic Diseases Group (GEAS) of the Spanish Society of Internal Medicine. The registry includes patients with sarcoidosis evaluated in Internal Medicine departments and specialised sarcoidosis units across Spain. Since its establishment in 2016, 36 hospitals have contributed data, including retrospective data from patients evaluated before registry creation and prospectively recorded data thereafter. Each participating centre updates its database annually; data extraction for the present analysis was completed in January 2026.

The study protocol was approved by the Clinical Research Ethics Committee of Hospital Clínic de Barcelona (coordinating centre) on 31 March 2016 (HCB/2016/0181) and was conducted in accordance with the principles of the amended Declaration of Helsinki. Written informed consent was obtained from all patients included in the prospective phase of the registry (from 31 March 2016 onwards). Consent was obtained by the treating physician at each participating centre at the time of registry enrolment using a standardised consent form approved by the Ethics Committee. For patients included in the retrospective phase, the requirement for informed consent was waived by the Ethics Committee, as data were obtained from pre-existing medical records. All data were pseudonymised prior to analysis.

### 2.2. Study Population

Adult patients aged ≥18 years with a confirmed diagnosis of sarcoidosis who were consecutively evaluated at the participating centres were eligible for inclusion. Diagnosis was established according to the joint recommendations of the American Thoracic Society (ATS), the European Respiratory Society (ERS), and WASOG criteria, requiring (1) compatible clinical and/or radiological findings; (2) histopathological evidence of non-caseating granulomas; and (3) exclusion of alternative causes of granulomatous disease. Patients fulfilling criteria (1) and (3) could also be included, provided that the reason for not meeting criterion (2) was specified.

### 2.3. Data Collection and Definitions

Data were collected retrospectively using a standardised electronic case report form. Variables were categorised into demographic characteristics and clinical manifestations, including pulmonary involvement and extrapulmonary organ involvement. All variables were defined in accordance with previously established criteria [4,23]. Ethnicity was classified using FDA-defined categories for descriptive analyses and dichotomised as White versus non-White for comparative analyses, reflecting the predominance of White patients in the cohort and the limited representation of other ethnic groups. 

Asymptomatic diagnosis was defined as an incidental diagnosis in the absence of any symptoms.

EOS was defined as a diagnosis of sarcoidosis established at ≥65 years of age, in line with the threshold most consistently adopted in previous studies [14,15,16,17,18]; patients diagnosed before 65 years were classified as non-EOS. Age at diagnosis was defined as the age at which the diagnosis of sarcoidosis was established and was used as the reference time point for all age-related analyses.

Pulmonary involvement was defined at diagnosis using a CT-based adaptation of the Scadding staging system based on high-resolution computed tomography (CT). Thoracic disease was classified into five stages: stage 0 (normal imaging), stage I (bilateral hilar lymphadenopathy [BHL] without pulmonary infiltrates), stage II (BHL with parenchymal infiltrates or interstitial changes), stage III (parenchymal infiltrates without BHL), and stage IV (advanced pulmonary fibrosis characterised by volume loss, reticular opacities, traction bronchiectasis, or honeycombing).

Extrapulmonary involvement was assessed according to the 2014 WASOG criteria [23] and was attributed to sarcoidosis only after reasonable exclusion of alternative causes, including comorbidities and concomitant medications. The following organ systems were evaluated: skin (including lupus pernio, papular or plaque lesions, and erythema nodosum); extrathoracic lymph nodes (enlargement confirmed by imaging or biopsy); ocular (uveitis, conjunctival granulomas, or optic nerve involvement); salivary glands (parotid or submandibular gland enlargement); hepatic (imaging abnormalities, laboratory alterations, or histological confirmation); splenic (imaging abnormalities and/or histological confirmation); renal (treatment-responsive renal failure or histological confirmation); calcium metabolism and vitamin D dysregulation (hypercalcaemia, hypercalciuria, or nephrolithiasis with suppressed PTH and elevated or normal 1,25-dihydroxyvitamin D); articular and bone (typical radiographic features, dactylitis, nodular tenosynovitis, or scintigraphic/PET uptake); muscular (MRI abnormalities, scintigraphic uptake, palpable muscle masses, or elevated muscle enzymes); bone marrow (diffuse PET uptake or unexplained cytopenias); ear, nose, and throat (granulomatous involvement on direct laryngoscopy, compatible imaging, or biopsy); neurologic (cranial neuropathies, central or peripheral nervous system involvement, or cerebrospinal fluid findings consistent with sarcoidosis); and cardiac (arrhythmias or conduction abnormalities and/or diagnostic evidence from cardiac magnetic resonance imaging, positron emission tomography, or biopsy). Organ involvement was recorded as present when WASOG criteria were met. The diagnostic work-up was performed according to routine clinical practice rather than a study-specific protocol; therefore, involvement not documented in the medical records was coded as absent, and documented absence could not be distinguished from lack of assessment. 

### 2.4. Statistical Analysis

All statistical analyses were performed using SPSS version 25 (IBM Corp., Armonk, NY, USA) and R version 4.5.2 (R Foundation for Statistical Computing, Vienna, Austria) via RStudio 2026.01.0 (RStudio, PBC, Boston, MA, USA).

Continuous variables were evaluated for normality using histograms, Q-Q plots, and the Shapiro–Wilk test. Normally distributed variables are presented as mean ± standard deviation, whereas non-normally distributed variables are presented as median and interquartile range (IQR). Categorical variables are presented as absolute frequencies and percentages. Sex, ethnicity, age at diagnosis, and CT-based Scadding stage were available for all patients; therefore, all analyses included the full cohort, and no imputation was performed.

Baseline characteristics were compared between EOS and non-EOS patients. Categorical variables were compared between groups using univariate logistic regression, and the overall distribution of CT-based Scadding stages was compared using the chi-square test.

In addition, median age at diagnosis was explored across demographic categories, CT-based Scadding stages, and extrapulmonary manifestations as an initial unadjusted analysis prior to multivariable modelling using the Mann–Whitney U test for comparisons between two groups. The overall distribution of age at diagnosis across CT-based Scadding stages was compared using the Kruskal–Wallis test, and each stage was subsequently compared with stage 0.

Given that age at diagnosis was the main exposure of interest, two complementary multivariable approaches were applied.

First, to identify baseline characteristics independently associated with EOS, multivariable logistic regression was performed. Variables of established clinical relevance (sex, ethnicity, and CT-based Scadding stage) were included a priori. Additional variables were selected based on univariate analysis (*p* < 0.20). Adjusted odds ratios (OR) with 95% confidence intervals (CI) were reported. Model calibration was assessed using the Hosmer–Lemeshow test and discrimination using Harrell’s C-statistic. Internal validation was performed with 200 bootstrap replications to estimate optimism-corrected C-statistics. Overall fit was evaluated with Nagelkerke R^2^. Multicollinearity and model stability were assessed using variance inflation factors (VIF > 10) and the number of events per model parameter (EPV ≥ 10), respectively. When the EPV was <10, the model was additionally fitted using Firth’s penalised likelihood regression, with profile penalised-likelihood confidence intervals, as a sensitivity analysis to reduce small-sample bias. Adjusted OR from the final model were displayed in forest plots.

Second, multivariable linear regression modelling was undertaken to explore independent associations between baseline characteristics and age at diagnosis treated as a continuous dependent variable. The same variable selection strategy described above was applied. Regression coefficients (β) with corresponding 95% CI were reported, representing the mean change in age at diagnosis associated with each variable. Model fit was assessed using the coefficient of determination (R^2^) and its adjusted counterpart. Model stability was additionally evaluated through the ratio of observations to model parameters (OVR; threshold ≥ 10).

Effect modification by sex was formally assessed in the full cohort by fitting multivariable logistic and linear regression models including all baseline variables and all sex × variable interaction terms simultaneously.

Additionally, to explore potential differences between men and women, univariate and multivariable regression analyses for both EOS and age at diagnosis (as a continuous variable) were repeated after stratifying the cohort by sex. Sex-specific multivariable models were constructed using the same variable selection strategy as in the main analyses. 

Sensitivity analyses were performed using alternative EOS cut-offs of 60 and 70 years, repeating the univariate and multivariable logistic models, overall and by sex, with the same covariates as the main models.

Centre and temporal effects were assessed using mixed-effects models with the centre as a random intercept, quantified by the intraclass correlation coefficient (ICC), and additionally adjusted for year of diagnosis.

To explore non-linear associations, age was modelled using restricted cubic splines in logistic regression models adjusted for sex and ethnicity restricted to the extrapulmonary manifestations independently associated with both EOS and age at diagnosis (cutaneous, articular/bone, neurological, and renal). Standardised adjusted predicted probabilities across age were estimated overall and by sex. Overall association and non-linearity were assessed using likelihood-ratio tests, and 95% CIs were obtained by bootstrap (200 iterations).

All statistical tests were two-tailed, and a *p* value < 0.05 was considered statistically significant.

### 2.5. Generative Artificial Intelligence Statement

During manuscript preparation, generative artificial intelligence tools were used to assist with language editing and improvement of grammar and style. No generative AI tools were used for study design, data collection, data analysis, or interpretation of results. The authors reviewed and edited all AI-assisted content and take full responsibility for the final manuscript.

## 3. Results

### 3.1. Baseline Characteristics of the Overall Cohort

Baseline demographic and clinical characteristics of the overall cohort are summarised in Table 1.

**Figure 1:**
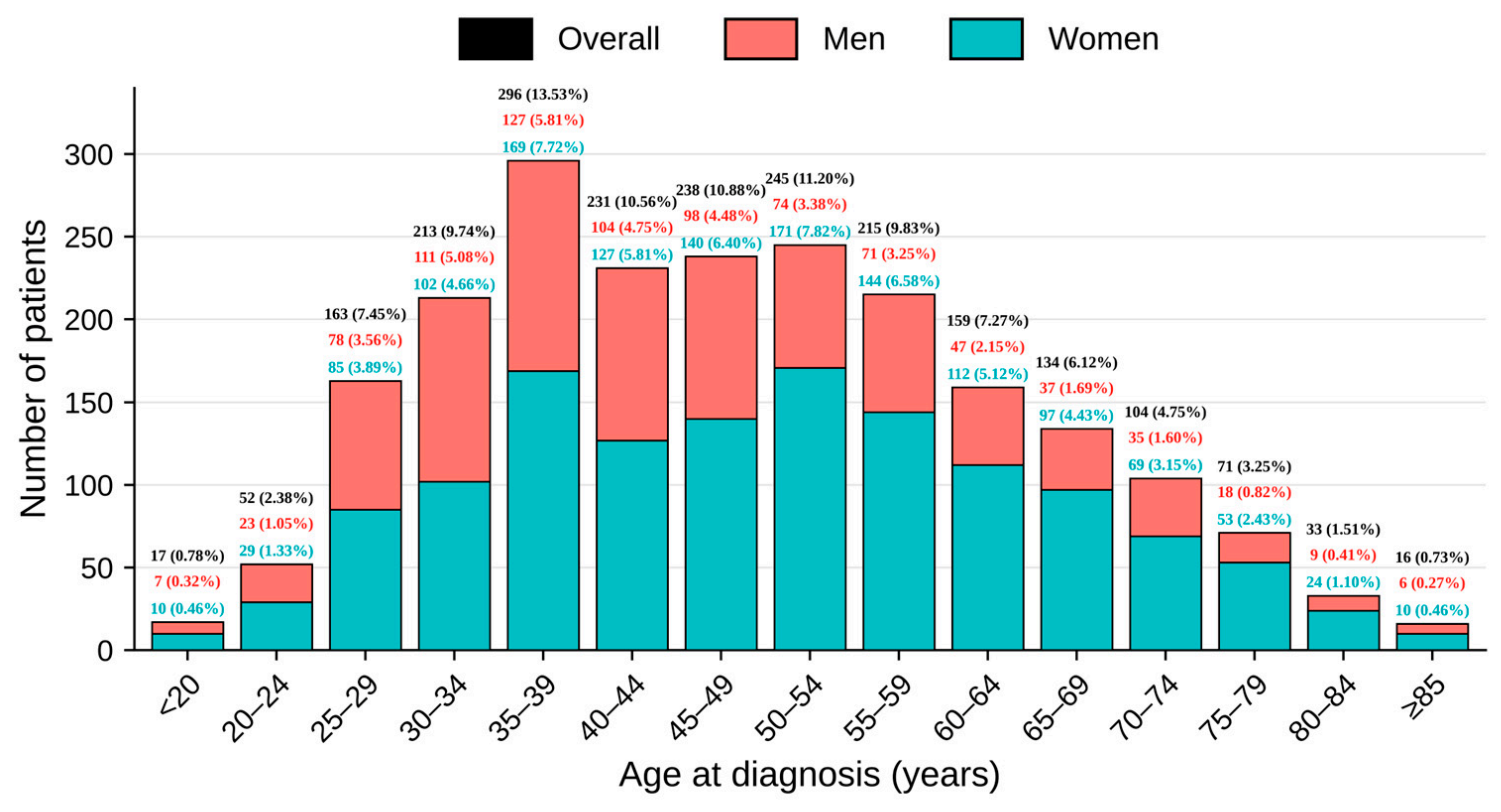
Sex-stratified age distribution of sarcoidosis at diagnosis.

A total of 2187 patients fulfilling the inclusion criteria were included in the study. Women accounted for 61.36% of the cohort (*n* = 1342), and men for 38.64% (*n* = 845). The mean age at diagnosis was 48.6 ± 15.2 years. The age distribution at diagnosis across predefined age groups is shown in Figure 1.

Ethnicity was predominantly White (88.07%), whereas 11.93% of patients were classified as non-White, including Hispanic (6.63%), Black (3.48%), and Asian (1.83%) individuals.

Pulmonary involvement was present in 89.12% of patients. The CT-based Scadding scale showed that stage II was the most frequent pattern (41.75%), followed by stage I (31.18%), stage III (13.58%) and stage IV (2.61%). In patients with stage IV disease, the most frequent fibrotic findings were bronchiectasis (75.44%), upper-lobe fibrocystic changes (36.84%), ground glass opacities (28.07%), and basal subpleural honeycombing (12.28%). 

Extrapulmonary involvement was identified in 1622 patients (74.17%). The skin was the most frequently affected organ (34.98%), mainly as erythema nodosum in 55.42% and lupus pernio in 14.64%, followed by extrathoracic lymph nodes (25.56%) and hepatic involvement (12.76%), with elevated liver enzymes ≥ 3 times the upper limit of normal in 43.73% and hepatic nodules in 25.81%. Ocular disease (11.98%) consisted mainly of uveitis (72.52%). Abnormalities in calcium metabolism (9.69%) included hypercalcaemia (59.91%), hypercalciuria (40.57%), and nephrolithiasis (16.04%). Splenic (8.92%), articular or bone (8.60%), neurological (7.09%), and renal involvement (3.80%) were less frequent. Articular or bone involvement was mainly identified by abnormal uptake on scintigraphy or PET (42.02%) and typical radiographic abnormalities (30.85%), and neurological involvement corresponded predominantly to granulomatous central nervous system disease (46.45%). Histological confirmation was obtained in 71.08% of patients with renal, 45.10% with cutaneous, 30.47% with hepatic, 12.26% with neurological, and 11.28% with splenic involvement.

### 3.2. Baseline Characteristics and Factors Independently Associated with Elderly-Onset Sarcoidosis

Baseline demographic and clinical characteristics according to EOS are summarised in Table 1.

Among the 2187 included patients, 358 (16.37%) met criteria for EOS. EOS patients were more frequently female (70.67% vs. 59.54%, *p* < 0.001) and of White ethnicity (97.21% vs. 86.28%, *p* < 0.001). The distribution of CT-based Scadding stages differed between groups (*p* = 0.026), with less stage II and more stage III in EOS, although no individual stage differed from stage 0. In stage IV disease, CT findings in EOS vs. non-EOS patients were bronchiectasis (92.31% vs. 70.45%), upper-lobe fibrocystic changes (38.46% vs. 36.36%), ground glass opacities (38.46% vs. 25.00%), and basal subpleural honeycombing (23.08% vs. 9.09%).

Regarding extrapulmonary manifestations, EOS patients had lower frequencies of cutaneous (23.18% vs. 37.29%, *p* < 0.001), articular or bone (3.35% vs. 9.62%, *p* < 0.001), and neurological involvement (4.19% vs. 7.65%, *p* = 0.022), while renal involvement was more frequent (7.26% vs. 3.12%, *p* < 0.001). In EOS vs. non-EOS patients, cutaneous involvement was mainly erythema nodosum (49.40% vs. 56.16%). Articular or bone involvement mainly comprised typical radiographic abnormalities (50.00% vs. 29.55%) and abnormal uptake on scintigraphy or PET (41.67% vs. 42.05%). Neurological involvement corresponded to peripheral neuropathy (33.33% vs. 20.71%) and granulomatous central nervous system disease (26.67% vs. 48.57%). Renal involvement was biopsy-proven in 73.08% vs. 70.18%.

**Figure 2:**
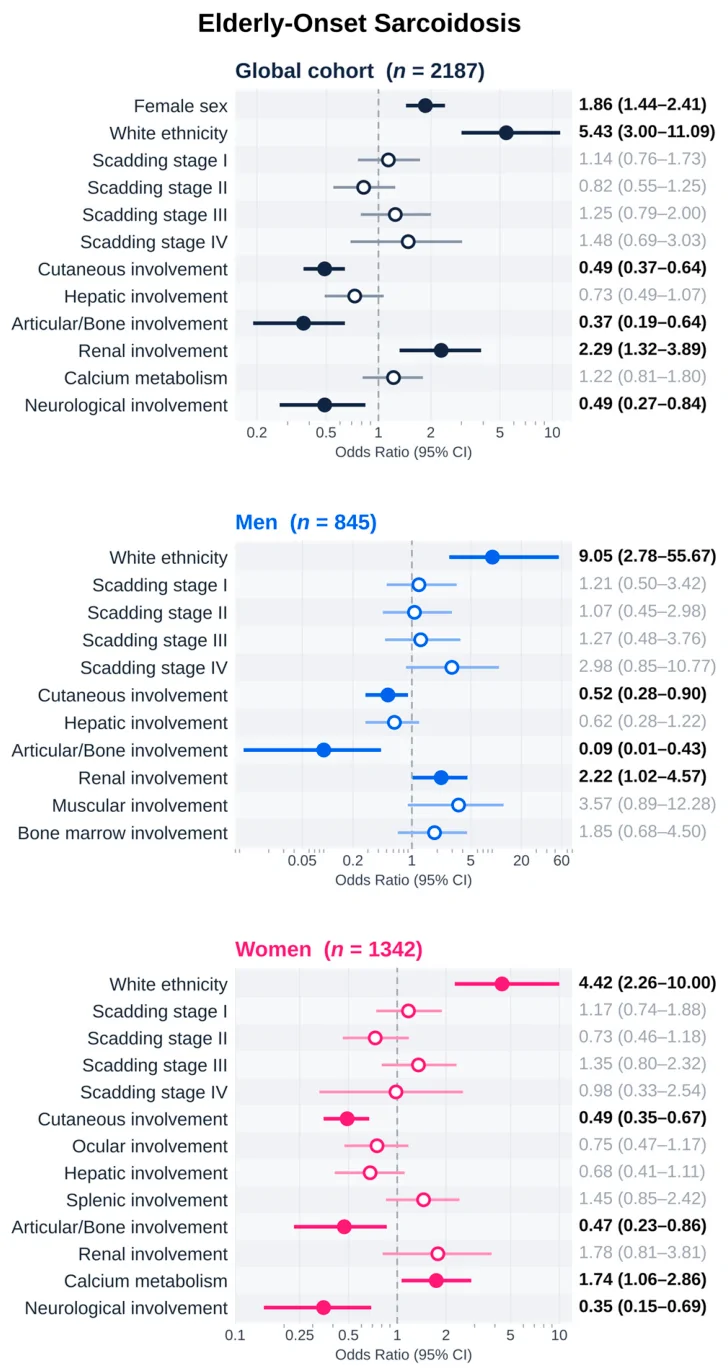
Combined forest plot of independent predictors of elderly-onset sarcoidosis (EOS) derived from multivariable logistic regression: overall cohort (black), men (blue), and women (red). Filled circles represent statistically significant associations (*p* < 0.05); open circles represent non-significant associations. Horizontal lines indicate 95% confidence intervals. The vertical dashed line denotes OR = 1. Reference category for CT-based Scadding stage: stage 0; reference category for ethnicity: non-White; reference category for sex: male.

In the multivariable logistic regression analysis (Figure 2), female sex (OR 1.86, 95% CI 1.44–2.41, *p* < 0.001), White ethnicity (OR 5.43, 95% CI 3.00–11.09, *p* < 0.001), and renal involvement (OR 2.29, 95% CI 1.32–3.89, *p* = 0.002) were independently associated with EOS. In contrast, cutaneous involvement (OR 0.49, 95% CI 0.37–0.64, *p* < 0.001), articular or bone disease (OR 0.37, 95% CI 0.19–0.64, *p* = 0.001), and neurological involvement (OR 0.49, 95% CI 0.27–0.84, *p* = 0.013) were independently associated with non-EOS patients.

The EPV, calculated per model parameter, was 29.8 for the overall model, 9.5 for men, and 19.5 for women. The Hosmer–Lemeshow test yielded χ^2^ (8) = 6.39 (*p* = 0.603), χ^2^ (8) = 10.39 (*p* = 0.245), and χ^2^ (8) = 8.36 (*p* = 0.399) for the overall, male, and female models, respectively. The apparent C-statistic was 0.681, 0.701, and 0.678, and the optimism-corrected C-statistic (200 bootstrap replications) was 0.668 (95% CI 0.656–0.708), 0.668 (95% CI 0.653–0.744), and 0.658 (95% CI 0.641–0.711) for the overall, male, and female models, respectively. Nagelkerke R^2^ values were 0.106, 0.119, and 0.105.

Sensitivity analyses using mixed-effects logistic regression showed minimal between-centre variability (ICC ≤ 5%). Additional adjustment for year of diagnosis did not materially modify the magnitude, direction, or statistical significance of the main associations.

Sex-stratified multivariable analyses identified both shared and sex-specific associations, which are summarised in Table 2.

In sex-stratified multivariable analyses, additional patterns were identified (Figure 2).

In the full cohort, a significant sex × variable interaction was observed only for calcium metabolism abnormalities (*p* for interaction = 0.045; Table 2).

In men, White ethnicity (OR 9.05, 95% CI 2.78–55.67, *p* = 0.002) and renal involvement (OR 2.22, 95% CI 1.02–4.57, *p* = 0.036) were independently associated with EOS, whereas cutaneous (OR 0.52, 95% CI 0.28–0.90, *p* = 0.025) and articular or bone involvement (OR 0.09, 95% CI 0.01–0.43, *p* = 0.019) were associated with non-EOS patients.

In women, White ethnicity (OR 4.42, 95% CI 2.26–10.00, *p* < 0.001) and abnormalities in calcium metabolism (OR 1.74, 95% CI 1.06–2.86, *p* = 0.032) were independently associated with EOS. In contrast, cutaneous (OR 0.49, 95% CI 0.35–0.67, *p* < 0.001), articular or bone (OR 0.47, 95% CI 0.23–0.86, *p* = 0.021), and neurological involvement (OR 0.35, 95% CI 0.15–0.69, *p* = 0.006) were associated with non-EOS patients.

As the EPV of the male model was below 10, a sensitivity analysis using Firth’s penalised regression was performed. All associations retained their direction and statistical significance, including cutaneous (OR 0.54, 95% CI 0.30–0.92) and renal involvement (OR 2.21, 95% CI 1.04–4.48). As expected, estimates based on few events were attenuated, including White ethnicity (OR 7.08, 95% CI 2.40–34.38) and articular or bone involvement (OR 0.14, 95% CI 0.02–0.53).

Sensitivity analyses using alternative cut-offs of 60 and 70 years to define EOS are presented in Appendix A (Table A1, Table A2, Table A3 and Table A4).

### 3.3. Associations Between Age at Diagnosis and Baseline Clinical Characteristics

Univariate and multivariable analyses of age at diagnosis according to baseline characteristics, for the overall cohort and stratified by sex, are presented in Table 3 and Table 4, respectively. 

In univariate analyses, women were diagnosed at an older median age than men (50.15 vs. 43.97 years, *p* < 0.001), and White patients were older at diagnosis than non-White individuals (48.31 vs. 43.87 years, *p* < 0.001).

Age at diagnosis differed across CT-based Scadding stages (*p* = 0.003), mainly owing to a younger age at diagnosis in patients with stage II disease, which was the only stage that differed significantly from stage 0 (median age 45.87 years, *p* = 0.003). 

Extrapulmonary manifestations also differed by age at diagnosis. Cutaneous involvement (45.67 vs. 48.47 years, *p* < 0.001) and articular or bone disease (43.01 vs. 47.73 years, *p* = 0.001) were associated with younger age at diagnosis, whereas renal involvement was associated with older age at diagnosis (57.87 vs. 46.90 years, *p* < 0.001).

**Figure 3:**
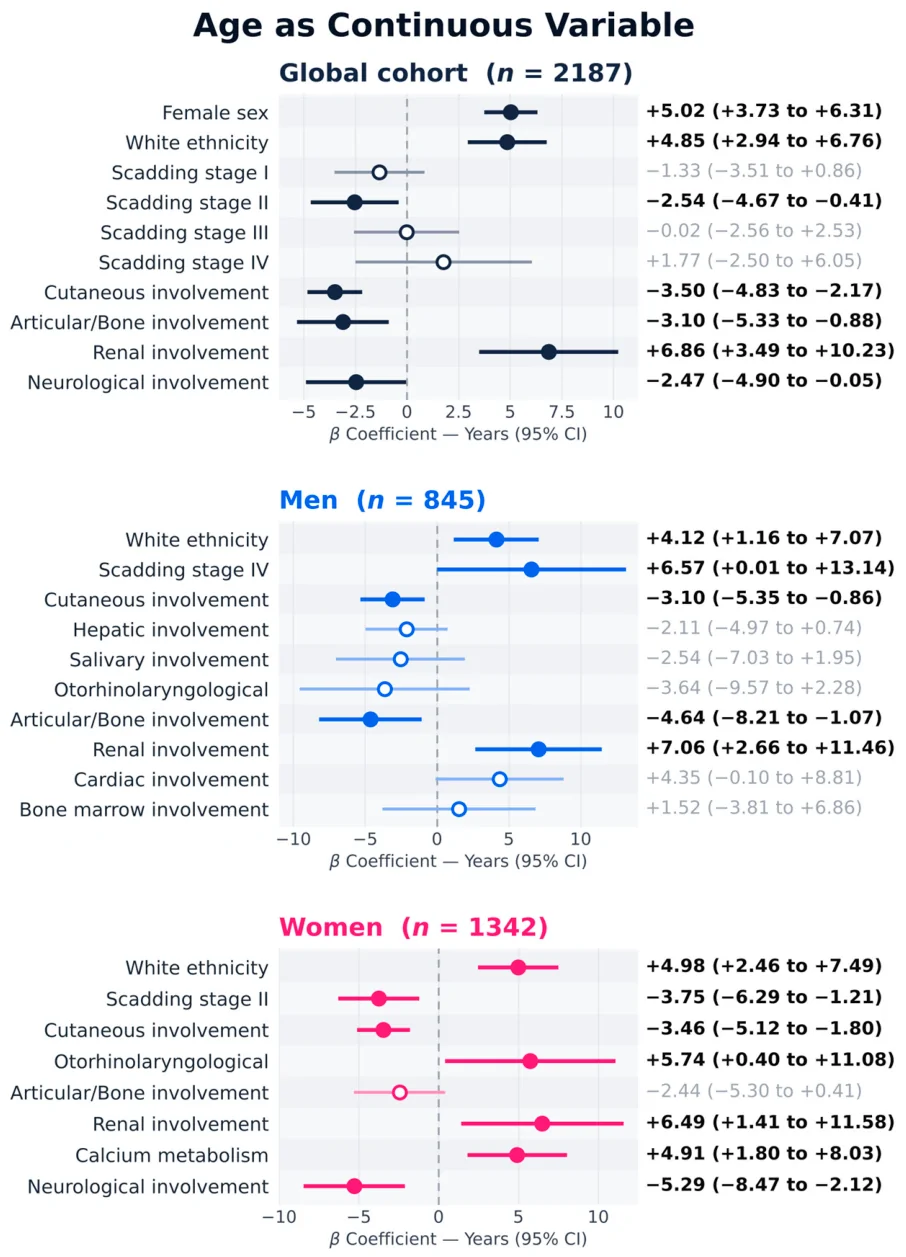
Combined forest plot of independent predictors of age at diagnosis (years) derived from multivariable linear regression: overall cohort (black), men (blue), and women (red). Filled circles represent statistically significant associations (*p* < 0.05); open circles represent non-significant associations. The β coefficient represents the mean difference in years of age at diagnosis associated with each variable. Horizontal lines indicate 95% confidence intervals. The vertical dashed line denotes β = 0. Reference category for CT-based Scadding stage: stage 0; reference category for ethnicity: non-White; reference category for sex: male.

In multivariable linear regression (Figure 3), female sex (β = +5.02, 95% CI 3.73–6.31, *p* < 0.001), White ethnicity (β = +4.85, 95% CI 2.94–6.76, *p* < 0.001), and renal involvement (β = +6.86, 95% CI 3.49–10.23, *p* < 0.001) were independently associated with older age at diagnosis. In contrast, CT-based Scadding stage II (β = −2.54, 95% CI −4.67 to −0.41, *p* = 0.020), cutaneous involvement (β = −3.50, 95% CI −4.83 to −2.17, *p* < 0.001), articular or bone disease (β = −3.10, 95% CI −5.33 to −0.88, *p* = 0.006), and neurological involvement (β = −2.47, 95% CI −4.90 to −0.05, *p* = 0.046) were independently associated with younger age at diagnosis. 

The OVR, calculated per model parameter, was 198.8 for the overall linear regression model, 65.0 for men, and 122.0 for women. The R^2^ values were 0.067, 0.060, and 0.064, and the adjusted R^2^ values were 0.061, 0.047, and 0.055 for the overall, male, and female models, respectively.

Similarly, sensitivity analyses using linear mixed-effects models showed minimal between-centre variability (ICC ≤ 5%), and additional adjustment for year of diagnosis did not materially alter the main associations.

Age at diagnosis according to baseline demographic, radiological, and extrapulmonary characteristics stratified by sex are displayed in Table 4.

In sex-stratified multivariable analyses, additional patterns were identified (Figure 3).

Formal tests of sex × variable interaction in the full cohort showed significant effect modification by sex for calcium metabolism abnormalities (*p* = 0.009), neurological involvement (*p* = 0.015), cardiac involvement (*p* = 0.021), ENT involvement (*p* = 0.022), and CT-based Scadding stages II (*p* = 0.037) and IV (*p* = 0.045) (Table 4).

In men, older age at diagnosis was associated with White ethnicity (β = +4.12, 95% CI 1.16–7.07, *p* = 0.006), renal involvement (β = +7.06, 95% CI 2.66–11.46, *p* = 0.002), and CT-based Scadding stage IV (β = +6.57, 95% CI 0.01–13.14, *p* = 0.049), while cutaneous (β = −3.10, 95% CI −5.35 to −0.86, *p* = 0.007) and articular or bone involvement (β = −4.64, 95% CI −8.21 to −1.07, *p* = 0.011) were associated with younger age at diagnosis.

In women, older age at diagnosis was associated with White ethnicity (β = +4.98, 95% CI 2.46–7.49, *p* < 0.001), renal involvement (β = +6.49, 95% CI 1.41–11.58, *p* = 0.012), calcium metabolism abnormalities (β = +4.91, 95% CI 1.80–8.03, *p* = 0.002), and ear–nose–throat (ENT) involvement (β = +5.74, 95% CI 0.40–11.08, *p* = 0.035). Conversely, CT-based Scadding stage II (β = −3.75, 95% CI −6.29 to −1.21, *p* = 0.004), cutaneous involvement (β = −3.46, 95% CI −5.12 to −1.80, *p* < 0.001), and neurological involvement (β = −5.29, 95% CI −8.47 to −2.12, *p* = 0.001) were independently associated with younger age at diagnosis.

**Figure A1:**
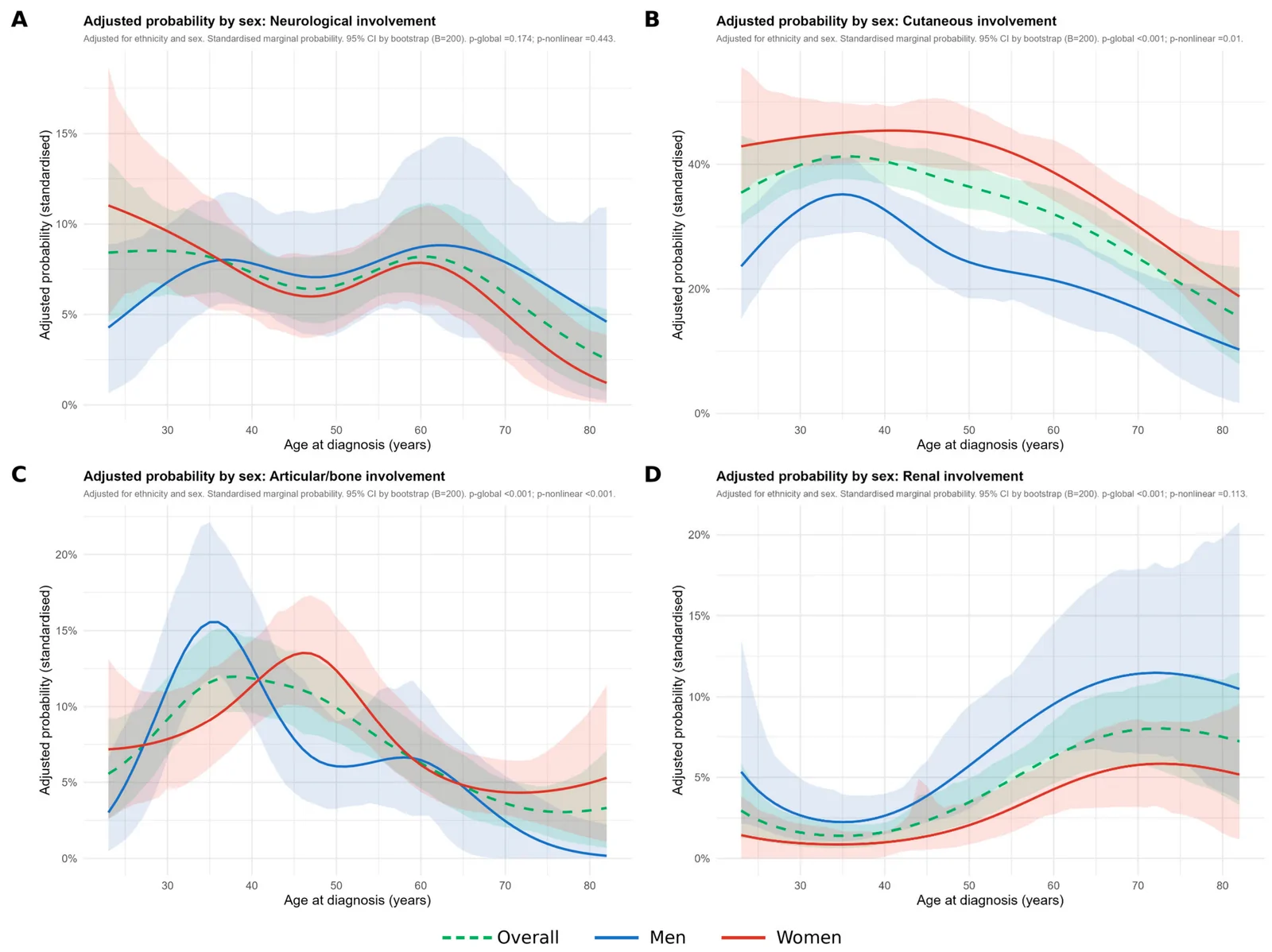
Overall and sex-stratified restricted cubic spline curves of adjusted predicted probability of selected extrapulmonary manifestations according to age at diagnosis: (**A**) neurological, (**B**) cutaneous, (**C**) articular/bone, and (**D**) renal involvement. Dashed green lines represent the overall cohort, blue lines men, and red lines women (common legend at the bottom). The *x*-axis shows age at diagnosis (years) and the *y*-axis the standardised adjusted predicted probability (%). Models were adjusted for sex and ethnicity and standardised to the observed covariate distribution. Shaded areas represent 95% confidence intervals estimated by bootstrap resampling (200 iterations). The *p* values for the overall association with age (p-global) and for non-linearity (p-nonlinear), derived from likelihood-ratio tests, are shown in each panel.

Non-linear associations between age at diagnosis and selected extrapulmonary manifestations were assessed using spline-based models. Significant overall associations with age were observed for cutaneous, articular or bone, and renal involvement (all *p*-global < 0.001), with evidence of non-linearity for cutaneous (*p*-nonlinear = 0.012) and articular or bone disease (*p*-nonlinear < 0.001) but not for renal involvement (*p*-nonlinear = 0.113). No significant age-dependent trajectory was identified for neurological involvement (*p*-global = 0.174). Adjusted probabilities of cutaneous and articular or bone involvement peaked in early to mid-adulthood and declined with increasing age, whereas the probability of renal involvement increased progressively from midlife onwards, reaching its highest values at older ages (Appendix B, Figure A1).

## 4. Discussion

In this large nationwide multicentre cohort, we evaluated the influence of age at diagnosis on the clinical phenotype of sarcoidosis, analysing age both categorically (EOS, which accounted for 16.37% of cases) and as a continuous variable. Across both approaches, older age at diagnosis was independently associated with female sex, White ethnicity, and renal involvement, whereas younger age at diagnosis was linked to cutaneous, articular/bone, and neurological manifestations. Exploratory sex-stratified analyses suggested additional differences. In women, calcium metabolism abnormalities and ENT involvement were more frequent at older ages at diagnosis. For pulmonary disease, stage II was associated with younger age at diagnosis in women and stage IV with older age in men. 

According to our series, EOS accounted for 16.37% of cases, which falls within the range reported in the literature (7–30%). These differences are likely explained by heterogeneity in age thresholds, populations, and study methodologies, as well as a growing recognition of sarcoidosis in older patients, with more recent studies reporting higher proportions and suggesting a shift toward increased diagnosis in this age group [6,14,15,16,17,18,20,21,22].

Female sex was independently associated with EOS and older age at diagnosis. The higher proportion of EOS among women in our cohort is consistent with the well-described second incidence peak in middle-aged and older women reported in large epidemiological studies [10,11,12,13]. These findings may reflect a potential contribution of sex-related biological factors to age-related differences in disease presentation [24,25], although the higher proportion of women in the older general population may also partly account for this association.

White ethnicity was also independently associated with EOS and older age at diagnosis in both the overall and sex-stratified models. Extensive European and North American data indicate earlier onset among Black and other non-White populations, with comparatively later presentation among White patients [5,7,26,27]. Although our cohort was predominantly White and non-White patients in Spain largely correspond to younger migrant populations, ethnicity remained independently associated with age at diagnosis in multivariable models, suggesting that ethnic differences in the timing of sarcoidosis onset may not simply reflect differences in clinical phenotype but could also be influenced by genetic, environmental, and socio-demographic factors [6,12,28].

With regard to pulmonary disease across the age spectrum, thoracic involvement remained highly prevalent across all age groups. In sex-stratified analyses, stage II was associated with younger age in women, consistent with the acute inflammatory presentations that more commonly occur in younger female patients, whereas stage IV was associated with older age at diagnosis in men. However, the latter finding was based on a small number of patients and reached only borderline significance and should therefore be interpreted with caution. These findings are consistent with previous reports showing that radiographic stage varies by age and sex [29,30,31].

Regarding extrapulmonary involvement, cutaneous manifestations were independently associated with younger age at diagnosis and were less frequent in EOS in both men and women. These findings are consistent with prior literature linking skin involvement to younger age at onset, as the full spectrum of cutaneous manifestations in sarcoidosis, including lupus pernio, maculopapular eruptions, subcutaneous nodules, scar sarcoidosis, and erythema nodosum in the context of Löfgren syndrome, tends to be attenuated in older-onset disease [14,15,16,17,32].

Articular and bone involvement was less frequent in EOS among both men and women. Previous studies have shown that musculoskeletal manifestations, including arthropathy, lytic bone lesions, and dactylitis, are more commonly observed in younger patients. Although some reports have described a higher overall frequency of articular and bone involvement in women, sex-related differences appear to be modest compared with the stronger association of age, and the heterogeneity of these manifestations may account for distinct age-related patterns [33,34,35,36]. 

Neurological involvement was associated with younger age at diagnosis and was less frequent in EOS. Sex-stratified analyses showed that this association was driven by women, with no significant relationship observed in men. These findings are consistent with previous reports describing a predominance of neurosarcoidosis in women and a mean age at diagnosis in the fourth to fifth decade, supporting its occurrence in younger patients. The predominance of neurological involvement in younger women raises the possibility that sex-related biological factors, including hormonal influences, may modulate susceptibility [37,38].

Neither salivary gland nor ENT involvement differed between EOS and non-EOS or across age in the overall analyses. However, sex-stratified analyses showed that ENT involvement was associated with older age at diagnosis in women. This finding is consistent with previous reports describing sinonasal sarcoidosis as a chronic phenotype with female predominance and onset at older ages [39]. Although these results should be interpreted cautiously given the low number of cases, they suggest that chronic extrapulmonary manifestations may be more frequent at older ages in women.

Renal involvement was associated with older age at diagnosis across analyses. Notably, more than 70% of renal cases were histologically confirmed, with a similar proportion in EOS and non-EOS patients, suggesting that this association is not solely explained by misattribution of age-related renal impairment. Renal sarcoidosis is most commonly driven by granulomatous interstitial nephritis (GIN), with subsequent chronic kidney dysfunction also well-established as a disease consequence [40,41,42].

The increased frequency of renal involvement with advancing age is particularly relevant. Older individuals have reduced renal functional reserve and a higher burden of comorbidities, factors that may unmask or amplify granulomatous or metabolic renal injury. Consequently, renal dysfunction in this population may be under-recognised or misattributed to age-related or comorbid conditions rather than sarcoidosis itself. Consistent with previous reports showing a higher prevalence of renal sarcoidosis in later adulthood [16,17,43,44], our findings reinforce the importance of maintaining a high index of suspicion and performing appropriate renal evaluation in older patients with sarcoidosis. 

Abnormalities in calcium metabolism were associated with EOS and with older age at diagnosis in women but not in men. Calcium–vitamin D homeostasis in sarcoidosis is closely linked to bone health, which may be particularly relevant in older women [45,46]. However, this association was based on a small number of events and was not corrected for multiple comparisons. It should therefore be interpreted as exploratory rather than as evidence of a sex-specific phenotype.

This study has several limitations inherent to its retrospective design. Organ involvement was not systematically assessed in all patients; the diagnostic work-up followed routine clinical practice; and organ-specific investigations were performed according to the treating physician’s clinical suspicion. Involvement not documented in the medical records was coded as absent, which may have led to misclassification, including, in particular, under-ascertainment of mild, subclinical, or organ-specific manifestations requiring targeted investigations. Residual differences in diagnostic pathways across age groups and centres cannot be excluded despite minimal between-centre variability. CT-based Scadding stages are not directly comparable with conventional chest radiograph-based staging given the higher sensitivity of CT. In addition, our analyses are based on age at diagnosis, which may not necessarily reflect age at disease onset. Furthermore, the absence of detailed data on treatment exposure, comorbidities, pulmonary function tests, and longitudinal outcomes limits causal and prognostic inference. Moreover, the sex-specific patterns observed in exploratory analyses should be interpreted with caution, as no formal correction for multiple comparisons was applied, and they require confirmation. Finally, the predominantly White composition of the cohort, which reflects the demographic profile of the Spanish population, may limit the generalisability of our findings to other ethnic groups, and the ethnic distribution of the cohort could not be directly compared with that of the general population. 

Despite these limitations, this study represents one of the largest nationwide multicentre cohorts examining age- and sex-related variation in sarcoidosis phenotype. The analysis of age both categorically and as a continuous variable, together with the use of standardised WASOG organ definitions and complementary analytical approaches, strengthens the robustness of the observed age-related patterns.

From a clinical perspective, these findings support incorporating age at diagnosis into the initial phenotypic assessment of sarcoidosis, recognising it as a variable consistently associated with disease expression. While comprehensive baseline organ evaluation remains essential, awareness of age-related patterns may help refine diagnostic suspicion and prioritise investigations. In older patients, renal function should be carefully assessed at diagnosis, and renal impairment should not be automatically attributed to age or comorbidities without considering sarcoidosis-related renal involvement. Overall, age emerges as a relevant factor associated with clinical presentation at diagnosis and should be considered when performing initial phenotypic stratification in sarcoidosis.

Future research should examine the prognostic and therapeutic implications of age-related phenotypes through prospective cohorts with longitudinal follow-up. Integrating comorbidity profiles, biomarkers, and mechanistic studies of immunosenescence will be key to clarifying whether EOS represents a distinct biological entity or an age-modified expression of disease and translating age-dependent patterns into clinically useful risk stratification tools.

## 5. Conclusions

In this large nationwide multicentre cohort, elderly-onset sarcoidosis accounted for 16.37% of cases. Age at diagnosis was associated with differences in clinical presentation both according to EOS status and across the age spectrum. Overall, older age at diagnosis and EOS were associated with female sex, White ethnicity, and renal involvement, whereas younger age at diagnosis was associated with cutaneous, articular/bone, and neurological manifestations.

Beyond these global patterns, exploratory sex-stratified analyses suggested additional differences: in women, calcium metabolism abnormalities were associated with both EOS and older age at diagnosis and ear–nose–throat involvement with older age. Regarding pulmonary involvement, CT-based Scadding stage II was associated with younger age at diagnosis, particularly in women, whereas in men CT-based Scadding stage IV was associated with older age at diagnosis. Further studies are needed to confirm these findings. 

## Figures and Tables

**Table 1:** Baseline demographic and clinical characteristics according to elderly-onset sarcoidosis.

	Univariate Analysis *				Multivariable Analysis †		
Variables	Total (*n*, %)	Non-EOS	EOS	*p* Value	OR	CI (95%)	*p* Value
	2187 (100%)	1829 (83.63%)	358 (16.37%)				
Sex							
Men	845 (38.64%)	740 (40.46%)	105 (29.33%)		Ref.		
Women	1342 (61.36%)	1089 (59.54%)	253 (70.67%)	<0.001	1.86	1.44–2.41	**<0.001**
Ethnicity							
Non-White	261 (11.93%)	251 (13.72%)	10 (2.79%)		Ref.		
White	1926 (88.07%)	1578 (86.28%)	348 (97.21%)	<0.001	5.43	3.00–11.09	**<0.001**
Asymptomatic diagnosis	117 (5.35%)	93 (5.08%)	24 (6.70%)	0.264			
CT-based Scadding scale				**0.026 ‡**			
0	238 (10.88%)	199 (10.88%)	39 (10.89%)	Ref.	Ref.		
I	682 (31.18%)	562 (30.73%)	120 (33.52%)	0.671	1.14	0.76–1.73	0.545
II	913 (41.75%)	788 (43.08%)	125 (34.92%)	0.290	0.82	0.55–1.25	0.344
III	297 (13.58%)	236 (12.90%)	61 (17.04%)	0.222	1.25	0.79–2.00	0.342
IV	57 (2.61%)	44 (2.41%)	13 (3.63%)	0.255	1.48	0.69–3.03	0.296
Extrapulmonary manifestations							
Cutaneous	765 (34.98%)	682 (37.29%)	83 (23.18%)	<0.001	0.49	0.37–0.64	**<0.001**
Extrathoracic lymph node	559 (25.56%)	465 (25.42%)	94 (26.26%)	0.741			
Ocular	262 (11.98%)	225 (12.30%)	37 (10.34%)	0.295			
Hepatic	279 (12.76%)	245 (13.40%)	34 (9.50%)	0.044	0.73	0.49–1.07	0.118
Splenic	195 (8.92%)	161 (8.80%)	34 (9.50%)	0.675			
Salivary	122 (5.58%)	103 (5.63%)	19 (5.31%)	0.807			
Otorhinolaryngological	55 (2.51%)	45 (2.46%)	10 (2.79%)	0.713			
Articular/Bone	188 (8.60%)	176 (9.62%)	12 (3.35%)	<0.001	0.37	0.19–0.64	**0.001**
Muscular	35 (1.60%)	29 (1.59%)	6 (1.68%)	0.901			
Renal	83 (3.80%)	57 (3.12%)	26 (7.26%)	<0.001	2.29	1.32–3.89	**0.002**
Calcium metabolism	212 (9.69%)	168 (9.19%)	44 (12.29%)	0.070	1.22	0.81–1.80	0.324
Neurological	155 (7.09%)	140 (7.65%)	15 (4.19%)	0.022	0.49	0.27–0.84	**0.013**
Cardiac	80 (3.66%)	69 (3.77%)	11 (3.07%)	0.520			
Bone marrow	72 (3.29%)	57 (3.12%)	15 (4.19%)	0.300			

EOS: elderly-onset sarcoidosis. * Univariate logistic regression (each category vs. reference). † Multivariable logistic regression; only variables selected via clinical relevance or univariate association (*p* < 0.20) are shown. ‡ Overall distribution, chi-square test. Bold *p* < 0.05.

**Table 2:** Baseline demographic and clinical characteristics according to EOS, stratified by sex.

Variables	Men (*n* = 845)							Women (*n* = 1342)							*p* for Interaction §
	Univariate Analysis *				Multivariable Analysis †			Univariate Analysis *				Multivariable Analysis †			
	Total (*n*, %)	Non-EOS	EOS	*p* Value	OR	CI (95%)	*p* Value	Total (*n*, %)	Non-EOS	EOS	*p* Value	OR	CI (95%)	*p* Value	
*n* (%)	845 (38.64%)	740 (87.57%)	105 (12.43%)					1342 (61.36%)	1089 (81.15%)	253 (18.85%)					
Ethnicity															
Non-White (ref.)	108 (12.78%)	106 (14.32%)	2 (1.90%)		Ref.			153 (11.40%)	145 (13.31%)	8 (3.16%)	Ref.	Ref.			
White	737 (87.22%)	634 (85.68%)	103 (98.10%)	0.003	9.05	2.78–55.67	**0.002**	1189 (88.60%)	944 (86.69%)	245 (96.84%)	<0.001	4.42	2.26–10.00	**<0.001**	0.317
Asymptomatic diagnosis															
	48 (5.68%)	40 (5.41%)	8 (7.62%)	0.489				69 (5.14%)	53 (4.87%)	16 (6.32%)	0.431				0.770
CT-based Scadding scale				0.242 ‡							**0.038 ‡**				
0 (ref.)	60 (7.10%)	54 (7.30%)	6 (5.71%)	Ref.	Ref.			178 (13.26%)	145 (13.31%)	33 (13.04%)	Ref.	Ref.			Ref.
I	258 (30.53%)	227 (30.68%)	31 (29.52%)	0.661	1.21	0.50–3.42	0.695	424 (31.59%)	335 (30.76%)	89 (35.18%)	0.495	1.17	0.74–1.88	0.506	0.879
II	380 (44.97%)	336 (45.41%)	44 (41.90%)	0.720	1.07	0.45–2.98	0.881	533 (39.72%)	452 (41.51%)	81 (32.02%)	0.293	0.73	0.46–1.18	0.189	0.376
III	120 (14.20%)	103 (13.92%)	17 (16.19%)	0.432	1.27	0.48–3.76	0.649	177 (13.19%)	133 (12.21%)	44 (17.39%)	0.150	1.35	0.80–2.32	0.264	0.971
IV	27 (3.20%)	20 (2.70%)	7 (6.67%)	0.062	2.98	0.85–10.77	0.086	30 (2.24%)	24 (2.20%)	6 (2.37%)	0.850	0.98	0.33–2.54	0.974	0.122
Extrapulmonary manifestations															
Cutaneous	227 (26.86%)	211 (28.51%)	16 (15.24%)	0.005	0.52	0.28–0.90	**0.025**	538 (40.09%)	471 (43.25%)	67 (26.48%)	<0.001	0.49	0.35–0.67	**<0.001**	0.874
Extrathoracic lymph node	196 (23.20%)	174 (23.51%)	22 (20.95%)	0.561				363 (27.05%)	291 (26.72%)	72 (28.46%)	0.575				0.567
Ocular	84 (9.94%)	74 (10.00%)	10 (9.52%)	0.879				178 (13.26%)	151 (13.87%)	27 (10.67%)	0.179	0.75	0.47–1.17	0.217	0.351
Hepatic	118 (13.96%)	108 (14.59%)	10 (9.52%)	0.164	0.62	0.28–1.22	0.195	161 (12.00%)	137 (12.58%)	24 (9.49%)	0.174	0.68	0.41–1.11	0.134	0.918
Splenic	88 (10.41%)	80 (10.81%)	8 (7.62%)	0.317				107 (7.97%)	81 (7.44%)	26 (10.28%)	0.135	1.45	0.85–2.42	0.167	0.071
Salivary	43 (5.09%)	40 (5.41%)	3 (2.86%)	0.275				79 (5.89%)	63 (5.79%)	16 (6.32%)	0.743				0.443
Otorhinolaryngological	24 (2.84%)	22 (2.97%)	2 (1.90%)	0.541				31 (2.31%)	23 (2.11%)	8 (3.16%)	0.320				0.194
Articular/bone	72 (8.52%)	71 (9.59%)	1 (0.95%)	0.018	0.09	0.01–0.43	**0.019**	116 (8.64%)	105 (9.64%)	11 (4.35%)	0.009	0.47	0.23–0.86	**0.021**	0.061
Muscular	16 (1.89%)	12 (1.62%)	4 (3.81%)	0.135	3.57	0.89–12.28	0.051	19 (1.42%)	17 (1.56%)	2 (0.79%)	0.359				0.055
Renal	46 (5.44%)	34 (4.59%)	12 (11.43%)	0.005	2.22	1.02–4.57	**0.036**	37 (2.76%)	23 (2.11%)	14 (5.53%)	0.004	1.78	0.81–3.81	0.142	0.447
Calcium metabolism	107 (12.66%)	94 (12.70%)	13 (12.38%)	0.926				105 (7.82%)	74 (6.80%)	31 (12.25%)	0.004	1.74	1.06–2.86	**0.032**	**0.045**
Neurological	62 (7.34%)	55 (7.43%)	7 (6.67%)	0.778				93 (6.93%)	85 (7.81%)	8 (3.16%)	0.011	0.35	0.15–0.69	**0.006**	0.081
Cardiac	43 (5.09%)	37 (5.00%)	6 (5.71%)	0.755				37 (2.76%)	32 (2.94%)	5 (1.98%)	0.403				0.136
Bone marrow	31 (3.67%)	24 (3.24%)	7 (6.67%)	0.088	1.85	0.68–4.50	0.194	41 (3.06%)	33 (3.03%)	8 (3.16%)	0.913				0.356

EOS: elderly-onset sarcoidosis. * Univariate logistic regression (each category vs. reference). † Multivariable logistic regression. ‡ Overall distribution, chi-square test. § Sex × variable interaction (multivariable logistic regression including all variables and interaction terms, whole cohort). Bold *p* < 0.05.

**Table 3:** Age at diagnosis according to baseline demographic, radiological, and extrapulmonary characteristics.

	Univariate Analysis *				Multivariable Analysis †		
Variables (*n*, %)		Median (Years)	IQR	*p* Value	Coeff. (β)	CI (95%)	*p* Value
Sex							
	Men (845, 38.64%)	43.97	34.60–55.99	Ref.	Ref.		
	Women (1342, 61.36%)	50.15	38.18–61.29	<0.001	+5.02	3.73–6.31	**<0.001**
Ethnicity							
	Non-White (261, 11.93%)	43.87	35.63–51.86	Ref.	Ref.		
	White (1926, 88.07%)	48.31	36.91–60.31	<0.001	+4.85	2.94–6.76	**<0.001**
Asymptomatic diagnosis (117, 5.35%)							
	Yes	51.02	38.27–61.47	0.174			
	No	47.36	36.62–59.05				
CT-based Scadding scale				**0.003 ‡**			
	0 (238, 10.88%)	51.50	37.99–60.05	Ref.	Ref.		
	I (682, 31.18%)	46.64	36.11–59.51	0.054	−1.33	−3.51–0.86	0.235
	II (913, 41.75%)	45.87	36.18–58.04	0.003	−2.54	−4.67–−0.41	**0.020**
	III (297, 13.58%)	49.88	38.01–61.05	0.822	−0.02	−2.56–2.53	0.991
	IV (57, 2.61%)	48.21	39.03–63.33	0.726	+1.77	−2.50–6.05	0.416
Extrapulmonary manifestations							
Cutaneous (765, 34.98%)	Yes	45.67	35.48–56.01	<0.001	−3.50	−4.83–−2.17	**<0.001**
	No	48.47	37.36–61.1				
Extrathoracic lymph node (559, 25.56%)	Yes	47.74	37.36–59.05	0.455			
	No	47.23	36.5–59.36				
Ocular (262, 11.98%)	Yes	47.73	37.54–60.04	0.720			
	No	47.35	36.62–59.16				
Hepatic (279, 12.76%)	Yes	46.59	37.00–58.25	0.459			
	No	47.55	36.62–59.55				
Splenic (195, 8.92%)	Yes	46.65	36.54–59.88	0.822			
	No	47.48	36.68–59.24				
Salivary (122, 5.58%)	Yes	45.57	36.41–59.40	0.691			
	No	47.48	36.67–59.26				
Otorhinolaryngological (55, 2.51%)	Yes	45.56	38.06–57.19	0.967			
	No	47.47	36.66–59.31				
Articular/bone (188, 8.60%)	Yes	43.01	36.16–52.71	0.001	−3.10	−5.33–−0.88	**0.006**
	No	47.73	36.7–59.94				
Muscular (35, 1.60%)	Yes	49.77	38.62–60.19	0.335			
	No	47.39	36.64–59.25				
Renal (83, 3.80%)	Yes	57.87	47.05–68.06	<0.001	+6.86	3.49–10.23	**<0.001**
	No	46.90	36.51–58.92				
Calcium metabolism (212, 9.69%)	Yes	50.52	38.54–63.03	0.020	+1.86	−0.33–4.04	0.095
	No	46.94	36.61–59.05				
Neurological (155, 7.09%)	Yes	45.44	36.41–57.80	0.146	−2.47	−4.90–−0.05	**0.046**
	No	47.55	36.67–59.54				
Cardiac (80, 3.66%)	Yes	48.63	40.66–59.71	0.384			
	No	47.38	36.61–59.23				
Bone marrow (72, 3.29%)	Yes	49.99	39.91–61.88	0.295			
	No	47.38	36.65–59.23				

IQR: interquartile range; β: regression coefficient. * Univariate analysis (Mann–Whitney U or Kruskal–Wallis). † Multivariable linear regression. ‡ Overall distribution, Kruskal–Wallis test. Bold *p* < 0.05.

**Table 4:** Age at diagnosis according to baseline demographic, radiological, and extrapulmonary characteristics, stratified by sex.

Variables	Men (*n* = 845)							Women (*n* = 1342)							*p* for Interaction §
	Univariate Analysis *				Multivariable Analysis †			Univariate Analysis *				Multivariable Analysis †			
		Median (Years)	IQR	*p* Value	Coeff. (β)	CI (95%)	*p* Value		Median (Years)	IQR	*p* Value	Coeff. (β)	CI (95%)	*p* Value	
Ethnicity															
Non-White		42.08	34.51–49.23	Ref.	Ref.				44.65	35.95–52.60	Ref.	Ref.			
White		44.03	34.70–57.39	0.036	+4.12	1.16–7.07	**0.006**		51.01	38.57–62.34	<0.001	+4.98	2.46–7.49	**<0.001**	0.769
Asymptomatic diagnosis															
	Yes	43.99	35.61–60.97	0.463				Yes	53.28	39.89–63.22	0.217				0.965
	No	43.97	34.60–55.89					No	50.11	38.05–61.17					
CT-based Scadding scale				0.389 ‡							**0.004 ‡**				
0		42.05	33.97–55.36	Ref.	Ref.				53.27	42.38–62.58	Ref.	Ref.			Ref.
I		44.07	33.84–55.85	0.623	+1.07	−2.99–5.14	0.605		49.52	37.20–62.18	0.070	−1.95	−4.56–0.66	0.142	0.158
II		43.06	35.03–54.92	0.658	+0.62	−3.33–4.56	0.759		48.25	37.36–59.50	0.003	−3.75	−6.29–−1.21	**0.004**	**0.037**
III		45.46	35.04–55.99	0.494	+0.88	−3.61–5.37	0.700		52.85	40.81–64.48	0.774	+0.46	−2.66–3.58	0.772	0.644
IV		46.39	38.47–65.54	0.077	+6.57	0.01–13.14	**0.049**		49.03	39.65–62.53	0.678	−1.45	−7.21–4.31	0.621	**0.045**
Extrapulmonary manifestations															
Cutaneous	Yes	40.03	33.69–50.52	0.001	−3.10	−5.35–−0.86	**0.007**	Yes	48.24	36.60–58.07	<0.001	−3.46	−5.12–−1.80	**<0.001**	0.831
	No	44.86	35.63–57.93					No	51.29	38.79–63.91					
Extrathoracic lymph node	Yes	43.76	34.67–54.02	0.426				Yes	51.01	39.62–61.63	0.273				0.926
	No	44.01	34.60–56.65					No	50.08	37.54–61.12					
Ocular	Yes	46.25	36.14–59.01	0.232				Yes	50.12	37.77–60.31	0.418				0.118
	No	43.55	34.47–55.85					No	50.18	38.34–61.49					
Hepatic	Yes	43.20	34.01–53.64	0.191	−2.11	−4.97–0.74	0.147	Yes	51.18	39.79–60.03	0.733				0.408
	No	44.06	34.73–56.30					No	50.11	37.93–61.42					
Splenic	Yes	44.19	33.94–52.67	0.360				Yes	51.74	38.78–64.86	0.284				0.266
	No	43.85	34.74–56.38					No	50.12	38.11–60.95					
Salivary	Yes	40.81	32.26–48.69	0.091	−2.54	−7.03–1.95	0.267	Yes	51.33	38.90–62.42	0.538				0.165
	No	44.04	34.72–56.41					No	50.12	38.14–61.06					
Otorhinolaryngological	Yes	40.32	31.39–46.75	0.108	−3.64	−9.57–2.28	0.228	Yes	52.12	44.72–65.60	0.141	+5.74	0.40–11.08	**0.035**	**0.022**
	No	44.03	34.75–56.26					No	50.14	38.07–61.13					
Articular/bone	Yes	38.49	33.70–46.00	0.005	−4.64	−8.21–−1.07	**0.011**	Yes	48.09	38.45–54.26	0.047	−2.44	−5.30–0.41	0.093	0.388
	No	44.45	34.76–56.90					No	50.30	38.18–62.02					
Muscular	Yes	51.22	34.03–63.97	0.282				Yes	49.77	39.69–59.28	0.757				0.363
	No	43.70	34.70–55.85					No	50.16	38.17–61.41					
Renal	Yes	52.79	42.65–64.96	0.001	+7.06	2.66–11.46	**0.002**	Yes	59.87	52.64–68.65	<0.001	+6.49	1.41–11.58	**0.012**	0.781
	No	43.36	34.45–55.60					No	49.94	38.04–60.95					
Calcium metabolism	Yes	43.97	34.18–54.93	0.950				Yes	56.29	48.39–67.18	<0.001	+4.91	1.80–8.03	**0.002**	**0.009**
	No	43.94	34.72–56.04					No	49.55	37.79–60.48					
Neurological	Yes	43.37	37.58–57.59	0.608				Yes	46.39	36.16–57.85	0.031	−5.29	−8.47–−2.12	**0.001**	**0.015**
	No	43.99	34.45–55.87					No	50.25	38.45–61.70					
Cardiac	Yes	48.00	42.82–59.46	0.021	+4.35	−0.10–8.81	0.056	Yes	48.94	40.42–59.86	0.695				**0.021**
	No	43.35	34.41–55.81					No	50.21	38.16–61.43					
Bone marrow	Yes	50.13	38.06–59.21	0.156	+1.52	−3.81–6.86	0.576	Yes	48.15	40.07–62.00	0.802				0.669
	No	43.64	34.58–55.78					No	50.16	38.16–61.17					

IQR: interquartile range; β: regression coefficient. * Univariate analysis (Mann–Whitney U or Kruskal–Wallis). † Multivariable linear regression. ‡ Overall distribution, Kruskal–Wallis test. § Sex × variable interaction (multivariable linear regression including all variables, whole cohort). Bold *p* < 0.05.

## Data Availability

The raw data supporting the conclusions of this article will be made available by the authors upon request.

## Abbreviations

The following abbreviations are used in this manuscript:

EOS	Elderly-Onset Sarcoidosis
Non-EOS	Sarcoidosis diagnosed at <65 years
OR	Odds Ratio
WASOG	World Association of Sarcoidosis and Other Granulomatous Disorders
GEAS	Autoimmune and Systemic Diseases Group
SARCOGEAS	Spanish Sarcoidosis Registry (derived from the GEAS group)
ATS	American Thoracic Society
ERS	European Respiratory Society
FDA	Food and Drug Administration
BHL	Bilateral Hilar Lymphadenopathy
CT	Computed Tomography
PTH	Parathyroid Hormone
PET	Positron Emission Tomography
MRI	Magnetic Resonance Imaging
SPSS	Statistical Package for the Social Sciences
RStudio	RStudio software environment
IQR	Interquartile Range
CI	Confidence Interval
VIF	Variance Inflation Factor
EPV	Events Per model Parameter
OVR	Observations-to-parameters ratio
β	Beta coefficient
ENT	Ear, Nose and Throat
GIN	Granulomatous Interstitial Nephritis
ICC	Intraclass Correlation Coefficient

## Appendix A

**Table A1:** Baseline demographic and clinical characteristics according to elderly-onset sarcoidosis defined with a cut-off of 60 years (sensitivity analysis).

	Univariate Analysis *				Multivariable Analysis †		
Variables	Total (*n*, %)	Non-EOS	EOS	*p* Value	OR	CI (95%)	*p* Value
	2187 (100%)	1670 (76.36%)	517 (23.64%)				
Sex							
Men	845 (38.64%)	693 (41.50%)	152 (29.40%)		Ref.		
Women	1342 (61.36%)	977 (58.50%)	365 (70.60%)	<0.001	1.90	1.52–2.38	**<0.001**
Ethnicity							
Non-White	261 (11.93%)	238 (14.25%)	23 (4.45%)		Ref.		
White	1926 (88.07%)	1432 (85.75%)	494 (95.55%)	<0.001	3.55	2.32–5.68	**<0.001**
CT-based Scadding scale							
0	238 (10.88%)	177 (10.60%)	61 (11.80%)	Ref.	Ref.		
I	682 (31.18%)	513 (30.72%)	169 (32.69%)	0.794	1.01	0.72–1.45	0.939
II	913 (41.75%)	722 (43.23%)	191 (36.94%)	0.118	0.80	0.57–1.14	0.219
III	297 (13.58%)	218 (13.05%)	79 (15.28%)	0.800	1.03	0.69–1.55	0.870
IV	57 (2.61%)	40 (2.40%)	17 (3.29%)	0.519	1.25	0.64–2.39	0.506
Extrapulmonary manifestations							
Cutaneous	765 (34.98%)	631 (37.78%)	134 (25.92%)	<0.001	0.55	0.44–0.69	**<0.001**
Extrathoracic lymph node	559 (25.56%)	426 (25.51%)	133 (25.73%)	0.921			
Ocular	262 (11.98%)	196 (11.74%)	66 (12.77%)	0.529			
Hepatic	279 (12.76%)	224 (13.41%)	55 (10.64%)	0.099	0.82	0.59–1.13	0.239
Splenic	195 (8.92%)	147 (8.80%)	48 (9.28%)	0.740			
Salivary	122 (5.58%)	93 (5.57%)	29 (5.61%)	0.972			
Otorhinolaryngological	55 (2.51%)	42 (2.51%)	13 (2.51%)	1.000			
Articular/bone	188 (8.60%)	167 (10.00%)	21 (4.06%)	<0.001	0.42	0.25–0.65	**<0.001**
Muscular	35 (1.60%)	26 (1.56%)	9 (1.74%)	0.771			
Renal	83 (3.80%)	48 (2.87%)	35 (6.77%)	<0.001	2.48	1.50–4.06	**<0.001**
Calcium metabolism	212 (9.69%)	153 (9.16%)	59 (11.41%)	0.131	1.11	0.78–1.58	0.549
Neurological	155 (7.09%)	125 (7.49%)	30 (5.80%)	0.194	0.72	0.46–1.09	0.131
Cardiac	80 (3.66%)	62 (3.71%)	18 (3.48%)	0.807			
Bone marrow	72 (3.29%)	52 (3.11%)	20 (3.87%)	0.402			

EOS: elderly-onset sarcoidosis, defined in this sensitivity analysis as age at diagnosis ≥ 60 years. * Univariate logistic regression. † Multivariable logistic regression including the same covariates as the main model (Table 1). Bold *p* < 0.05.

**Table A2:** Baseline demographic and clinical characteristics according to elderly-onset sarcoidosis defined with a cut-off of 60 years, stratified by sex (sensitivity analysis).

Variables	Men (*n* = 845)							Women (*n* = 1342)						
	Univariate Analysis *				Multivariable Analysis †			Univariate Analysis *				Multivariable Analysis †		
	Total (*n*, %)	Non-EOS	EOS	*p* Value	OR	CI (95%)	*p* Value	Total (*n*, %)	Non-EOS	EOS	*p* Value	OR	CI (95%)	*p* Value
*n* (%)	845 (38.64%)	693 (82.01%)	152 (17.99%)					1342 (61.36%)	977 (72.80%)	365 (27.20%)				
Ethnicity														
Non-White (ref.)	108 (12.78%)	103 (14.86%)	5 (3.29%)		Ref.			153 (11.40%)	135 (13.82%)	18 (4.93%)	Ref.	Ref.		
White	737 (87.22%)	590 (85.14%)	147 (96.71%)	<0.001	5.39	2.36–15.56	**<0.001**	1189 (88.60%)	842 (86.18%)	347 (95.07%)	<0.001	2.94	1.80–5.07	**<0.001**
CT-based Scadding scale														
0 (ref.)	60 (7.10%)	52 (7.50%)	8 (5.26%)	Ref.	Ref.			178 (13.26%)	125 (12.79%)	53 (14.52%)	Ref.	Ref.		
I	258 (30.53%)	210 (30.30%)	48 (31.58%)	0.337	1.56	0.71–3.83	0.292	424 (31.59%)	303 (31.01%)	121 (33.15%)	0.760	0.96	0.64–1.43	0.821
II	380 (44.97%)	314 (45.31%)	66 (43.42%)	0.439	1.35	0.63–3.26	0.469	533 (39.72%)	408 (41.76%)	125 (34.25%)	0.093	0.69	0.47–1.03	0.064
III	120 (14.20%)	98 (14.14%)	22 (14.47%)	0.398	1.31	0.55–3.39	0.563	177 (13.19%)	120 (12.28%)	57 (15.62%)	0.621	1.08	0.67–1.72	0.761
IV	27 (3.20%)	19 (2.74%)	8 (5.26%)	0.076	2.65	0.83–8.57	0.097	30 (2.24%)	21 (2.15%)	9 (2.47%)	0.980	0.92	0.37–2.14	0.858
Extrapulmonary manifestations														
Cutaneous	227 (26.86%)	199 (28.72%)	28 (18.42%)	0.010	0.63	0.39–0.98	**0.046**	538 (40.09%)	432 (44.22%)	106 (29.04%)	<0.001	0.54	0.41–0.70	**<0.001**
Extrathoracic lymph node	196 (23.20%)	165 (23.81%)	31 (20.39%)	0.367				363 (27.05%)	261 (26.71%)	102 (27.95%)	0.652			
Ocular	84 (9.94%)	65 (9.38%)	19 (12.50%)	0.246				178 (13.26%)	131 (13.41%)	47 (12.88%)	0.798	0.96	0.66–1.39	0.839
Hepatic	118 (13.96%)	104 (15.01%)	14 (9.21%)	0.065	0.59	0.31–1.07	0.097	161 (12.00%)	120 (12.28%)	41 (11.23%)	0.599	0.86	0.56–1.29	0.466
Splenic	88 (10.41%)	77 (11.11%)	11 (7.24%)	0.159				107 (7.97%)	70 (7.16%)	37 (10.14%)	0.075	1.42	0.88–2.25	0.141
Salivary	43 (5.09%)	40 (5.77%)	3 (1.97%)	0.066				79 (5.89%)	53 (5.42%)	26 (7.12%)	0.241			
Otorhinolaryngological	24 (2.84%)	22 (3.17%)	2 (1.32%)	0.227				31 (2.31%)	20 (2.05%)	11 (3.01%)	0.297			
Articular/bone	72 (8.52%)	68 (9.81%)	4 (2.63%)	0.008	0.25	0.07–0.62	**0.008**	116 (8.64%)	99 (10.13%)	17 (4.66%)	0.002	0.46	0.26–0.78	**0.006**
Muscular	16 (1.89%)	11 (1.59%)	5 (3.29%)	0.173	2.78	0.79–8.83	0.090	19 (1.42%)	15 (1.54%)	4 (1.10%)	0.546			
Renal	46 (5.44%)	29 (4.18%)	17 (11.18%)	<0.001	2.80	1.41–5.47	**0.003**	37 (2.76%)	19 (1.94%)	18 (4.93%)	0.004	1.94	0.93–4.01	0.074
Calcium metabolism	107 (12.66%)	88 (12.70%)	19 (12.50%)	0.947				105 (7.82%)	65 (6.65%)	40 (10.96%)	0.010	1.47	0.92–2.32	0.105
Neurological	62 (7.34%)	51 (7.36%)	11 (7.24%)	0.958				93 (6.93%)	74 (7.57%)	19 (5.21%)	0.131	0.59	0.34–0.99	0.053
Cardiac	43 (5.09%)	34 (4.91%)	9 (5.92%)	0.607				37 (2.76%)	28 (2.87%)	9 (2.47%)	0.691			
Bone marrow	31 (3.67%)	24 (3.46%)	7 (4.61%)	0.499	1.05	0.39–2.50	0.924	41 (3.06%)	28 (2.87%)	13 (3.56%)	0.511			

EOS: elderly-onset sarcoidosis, defined in this sensitivity analysis as age at diagnosis ≥ 60 years. * Univariate logistic regression. † Multivariable logistic regression including the same covariates as the corresponding main sex-specific models (Table 2). Bold *p* < 0.05.

**Table A3:** Baseline demographic and clinical characteristics according to elderly-onset sarcoidosis defined with a cut-off of 70 years (sensitivity analysis).

	Univariate Analysis *				Multivariable Analysis †		
Variables	Total (*n*, %)	Non-EOS	EOS	*p* Value	OR	CI (95%)	*p* Value
	2187 (100%)	1963 (89.76%)	224 (10.24%)				
Sex							
Men	845 (38.64%)	777 (39.58%)	68 (30.36%)		Ref.		
Women	1342 (61.36%)	1186 (60.42%)	156 (69.64%)	0.008	1.67	1.23–2.29	**0.001**
Ethnicity							
Non-White	261 (11.93%)	253 (12.89%)	8 (3.57%)		Ref.		
White	1926 (88.07%)	1710 (87.11%)	216 (96.43%)	<0.001	3.75	1.94–8.41	**<0.001**
CT-based Scadding scale							
0	238 (10.88%)	209 (10.65%)	29 (12.95%)	Ref.	Ref.		
I	682 (31.18%)	606 (30.87%)	76 (33.93%)	0.664	0.91	0.57–1.47	0.683
II	913 (41.75%)	844 (43.00%)	69 (30.80%)	0.024	0.56	0.35–0.92	**0.018**
III	297 (13.58%)	256 (13.04%)	41 (18.30%)	0.581	1.04	0.62–1.77	0.885
IV	57 (2.61%)	48 (2.45%)	9 (4.02%)	0.467	1.24	0.51–2.76	0.619
Extrapulmonary manifestations							
Cutaneous	765 (34.98%)	720 (36.68%)	45 (20.09%)	<0.001	0.41	0.29–0.58	**<0.001**
Extrathoracic lymph node	559 (25.56%)	505 (25.73%)	54 (24.11%)	0.599			
Ocular	262 (11.98%)	241 (12.28%)	21 (9.38%)	0.207			
Hepatic	279 (12.76%)	259 (13.19%)	20 (8.93%)	0.072	0.69	0.41–1.10	0.138
Splenic	195 (8.92%)	178 (9.07%)	17 (7.59%)	0.461			
Salivary	122 (5.58%)	113 (5.76%)	9 (4.02%)	0.285			
Otorhinolaryngological	55 (2.51%)	48 (2.45%)	7 (3.12%)	0.539			
Articular/bone	188 (8.60%)	180 (9.17%)	8 (3.57%)	0.006	0.44	0.19–0.86	**0.027**
Muscular	35 (1.60%)	31 (1.58%)	4 (1.79%)	0.816			
Renal	83 (3.80%)	68 (3.46%)	15 (6.70%)	0.019	1.68	0.86–3.10	0.113
Calcium metabolism	212 (9.69%)	183 (9.32%)	29 (12.95%)	0.084	1.34	0.83–2.11	0.215
Neurological	155 (7.09%)	145 (7.39%)	10 (4.46%)	0.110	0.53	0.25–0.98	0.062
Cardiac	80 (3.66%)	73 (3.72%)	7 (3.12%)	0.654			
Bone marrow	72 (3.29%)	62 (3.16%)	10 (4.46%)	0.302			

EOS: elderly-onset sarcoidosis, defined in this sensitivity analysis as age at diagnosis ≥ 70 years. * Univariate logistic regression. † Multivariable logistic regression including the same covariates as the main model (Table 1). Bold *p* < 0.05.

**Table A4:** Baseline demographic and clinical characteristics according to elderly-onset sarcoidosis defined with a cut-off of 70 years, stratified by sex (sensitivity analysis).

Variables	Men (*n* = 845)							Women (*n* = 1342)						
	Univariate Analysis *				Multivariable Analysis †			Univariate Analysis *				Multivariable Analysis †		
	Total (*n*, %)	Non-EOS	EOS	*p* Value	OR	CI (95%)	*p* Value	Total (*n*, %)	Non-EOS	EOS	*p* Value	OR	CI (95%)	*p* Value
*n* (%)	845 (38.64%)	777 (91.95%)	68 (8.05%)					1342 (61.36%)	1186 (88.38%)	156 (11.62%)				
Ethnicity														
Non-White (ref.)	108 (12.78%)	106 (13.64%)	2 (2.94%)		Ref.			153 (11.40%)	147 (12.39%)	6 (3.85%)	Ref.	Ref.		
White	737 (87.22%)	671 (86.36%)	66 (97.06%)	0.023	5.44	1.65–33.62	**0.020**	1189 (88.60%)	1039 (87.61%)	150 (96.15%)	0.003	3.20	1.50–8.30	**0.007**
CT-based Scadding scale														
0 (ref.)	60 (7.10%)	56 (7.21%)	4 (5.88%)	Ref.	Ref.			178 (13.26%)	153 (12.90%)	25 (16.03%)	Ref.	Ref.		
I	258 (30.53%)	235 (30.24%)	23 (33.82%)	0.575	1.33	0.48–4.76	0.616	424 (31.59%)	371 (31.28%)	53 (33.97%)	0.607	0.84	0.50–1.44	0.513
II	380 (44.97%)	358 (46.07%)	22 (32.35%)	0.789	0.77	0.28–2.76	0.653	533 (39.72%)	486 (40.98%)	47 (30.13%)	0.047	0.53	0.31–0.91	**0.019**
III	120 (14.20%)	107 (13.77%)	13 (19.12%)	0.372	1.44	0.47–5.37	0.551	177 (13.19%)	149 (12.56%)	28 (17.95%)	0.639	0.98	0.53–1.80	0.935
IV	27 (3.20%)	21 (2.70%)	6 (8.82%)	0.046	3.64	0.92–15.97	0.069	30 (2.24%)	27 (2.28%)	3 (1.92%)	0.550	0.56	0.12–1.79	0.376
Extrapulmonary manifestations														
Cutaneous	227 (26.86%)	218 (28.06%)	9 (13.24%)	0.010	0.44	0.20–0.88	**0.030**	538 (40.09%)	502 (42.33%)	36 (23.08%)	<0.001	0.40	0.26–0.59	**<0.001**
Extrathoracic lymph node	196 (23.20%)	181 (23.29%)	15 (22.06%)	0.817				363 (27.05%)	324 (27.32%)	39 (25.00%)	0.540			
Ocular	84 (9.94%)	78 (10.04%)	6 (8.82%)	0.748				178 (13.26%)	163 (13.74%)	15 (9.62%)	0.155	0.65	0.35–1.13	0.145
Hepatic	118 (13.96%)	112 (14.41%)	6 (8.82%)	0.207	0.60	0.22–1.37	0.264	161 (12.00%)	147 (12.39%)	14 (8.97%)	0.219	0.70	0.37–1.26	0.263
Splenic	88 (10.41%)	83 (10.68%)	5 (7.35%)	0.390				107 (7.97%)	95 (8.01%)	12 (7.69%)	0.890	0.91	0.44–1.73	0.779
Salivary	43 (5.09%)	41 (5.28%)	2 (2.94%)	0.408				79 (5.89%)	72 (6.07%)	7 (4.49%)	0.431			
Otorhinolaryngological	24 (2.84%)	24 (3.09%)	0 (0.00%)	0.999				31 (2.31%)	24 (2.02%)	7 (4.49%)	0.061			
Articular/bone	72 (8.52%)	71 (9.14%)	1 (1.47%)	0.060	0.16	0.01–0.78	0.077	116 (8.64%)	109 (9.19%)	7 (4.49%)	0.055	0.55	0.23–1.14	0.143
Muscular	16 (1.89%)	14 (1.80%)	2 (2.94%)	0.513	2.42	0.36–9.93	0.273	19 (1.42%)	17 (1.43%)	2 (1.28%)	0.881			
Renal	46 (5.44%)	38 (4.89%)	8 (11.76%)	0.021	2.14	0.86–4.83	0.081	37 (2.76%)	30 (2.53%)	7 (4.49%)	0.166	1.16	0.42–2.81	0.759
Calcium metabolism	107 (12.66%)	97 (12.48%)	10 (14.71%)	0.598				105 (7.82%)	86 (7.25%)	19 (12.18%)	0.033	1.76	0.95–3.12	0.061
Neurological	62 (7.34%)	57 (7.34%)	5 (7.35%)	0.996				93 (6.93%)	88 (7.42%)	5 (3.21%)	0.059	0.37	0.13–0.84	**0.035**
Cardiac	43 (5.09%)	39 (5.02%)	4 (5.88%)	0.756				37 (2.76%)	34 (2.87%)	3 (1.92%)	0.501			
Bone marrow	31 (3.67%)	27 (3.47%)	4 (5.88%)	0.317	1.55	0.42–4.44	0.455	41 (3.06%)	35 (2.95%)	6 (3.85%)	0.543			

EOS: elderly-onset sarcoidosis, defined in this sensitivity analysis as age at diagnosis ≥ 70 years. * Univariate logistic regression. † Multivariable logistic regression including the same covariates as the corresponding main sex-specific models (Table 2). Bold *p* < 0.05.
